# *Candida* Infections, Causes, Targets, and Resistance Mechanisms: Traditional and Alternative Antifungal Agents

**DOI:** 10.1155/2013/204237

**Published:** 2013-06-26

**Authors:** Claudia Spampinato, Darío Leonardi

**Affiliations:** ^1^Departamento de Química Biológica, Facultad de Ciencias Bioquímicas y Farmacéuticas, Universidad Nacional de Rosario (UNR), Suipacha 531, 2000 Rosario, Argentina; ^2^Centro de Estudios Fotosintéticos y Bioquímicos (CEFOBI, UNR-CONICET), Suipacha 531, 2000 Rosario, Argentina; ^3^Departamento de Tecnología Farmacéutica, Facultad de Ciencias Bioquímicas y Farmacéuticas, Universidad Nacional de Rosario (UNR), Suipacha 531, 2000 Rosario, Argentina; ^4^Instituto de Química Rosario (IQUIR, UNR-CONICET), Suipacha 531, 2000 Rosario, Argentina

## Abstract

The genus *Candida* includes about 200 different species, but only a few species are human opportunistic pathogens and cause infections when the host becomes debilitated or immunocompromised. *Candida* infections can be superficial or invasive. Superficial infections often affect the skin or mucous membranes and can be treated successfully with topical antifungal drugs. However, invasive fungal infections are often life-threatening, probably due to inefficient diagnostic methods and inappropriate initial antifungal therapies. Here, we briefly review our current knowledge of pathogenic species of the genus *Candida* and yeast infection causes and then focus on current antifungal drugs and resistance mechanisms. An overview of new therapeutic alternatives for the treatment of *Candida* infections is also provided.

## 1. Introduction

*Candida albicans* is the most important fungal opportunistic pathogen. It usually resides as a commensal in the gastrointestinal and genitourinary tracts and in the oral and conjunctival flora [1–5]. However, it causes infection when the host becomes debilitated or immunocompromised. These infections can be superficial and affect the skin or mucous membrane [6] or can invade the bloodstream and disseminate to internal organs. Risk factors for invasive candidiasis include surgery (especially abdominal surgery), burns, long-term stay in an intensive care unit, and previous administration of broad-spectrum antibiotics and immunosuppressive agents [7–10]. Advances in medical management as antineoplasic chemotherapy, organ transplantation, hemodialysis, parenteral nutrition, and central venous catheters also contribute to fungal invasion and colonization [11]. Other *Candida* species found in healthy individuals include *Candida glabrata*,* Candida tropicalis*,* Candida parapsilosis, *and* Candida krusei* [12]. All five mentioned species cause more than 90% of invasive infections, although the relative prevalence of the species depends on the geographical location, patient population, and clinical settings [12–14]. Emergence of *Candida guilliermondii*, *Candida kefyr*, *Candida rugosa*, *Candida dubliniensis,* and* Candida famata *as pathogens has also been reported worldwide [6, 14]. In fact, the National Nosocomial Infections Surveillance System (NNISS) reports *Candida* species as the fourth most common nosocomial bloodstream pathogen [15]. Mortality rates are estimated to be as high as 45% [16], probably due to inefficient diagnostic methods and inappropriate initial antifungal therapies [17]. 

## 2. Antifungal Drugs in Clinical Treatments

**Figure 1:**
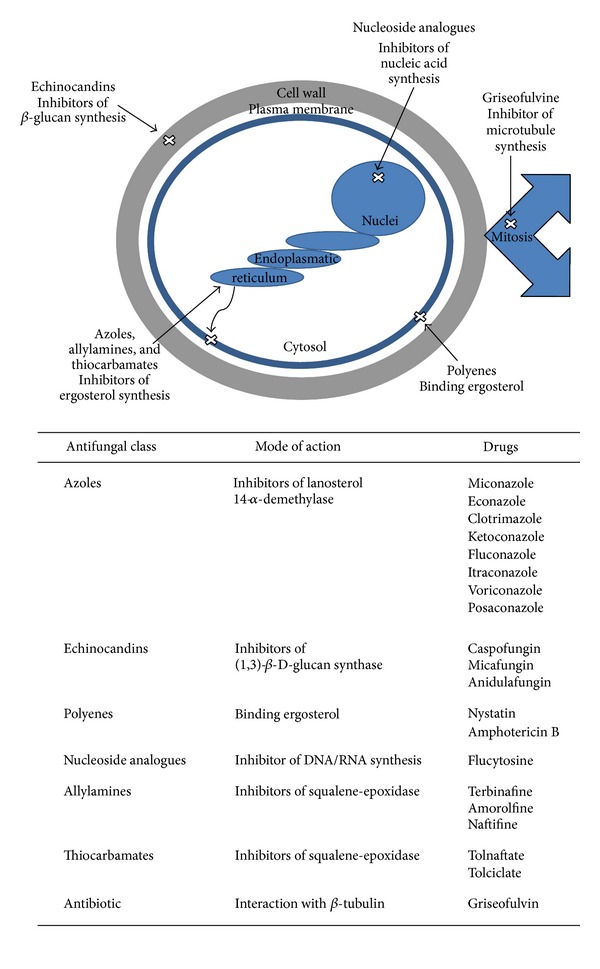
Primary targets and mode of action of several antifungal agents.

Although the antifungal drugs used in clinical treatments appear to be diverse and numerous, only few classes of antifungal agents are currently available to treat mucosal or systemic infections with *Candida* spp. (Figure 1) [18–20]. 

### 2.1. Azoles: Inhibitors of the Lanosterol 14-*α*-Demethylase

The largest family of antifungal drugs is the azole family. Azoles disrupt the cell membrane by inhibiting the activity of the lanosterol 14-*α*-demethylase [21], enzyme involved in the biosynthesis of ergosterol (Figure 1). Ergosterol, analogous to cholesterol in animal cells, is the largest sterol component of the fungal cell membrane. Since ergosterol and cholesterol have sufficient structural differences, most antifungal agents targeted to ergosterol binding or biosynthesis does not cross-react with host cells. The azole family includes imidazoles (miconazole, econazole, clotrimazole, and ketoconazole) and triazoles (fluconazole, itraconazole, and the latest agent voriconazole (second-generation, synthetic triazole derivative of fluconazole) and posaconazole (hydroxylated analogue of itraconazole)) [21, 22]. Many azoles are effective both for topical use and for the treatment and prophylaxis of invasive fungal infections [22]. In this regard, these agents have the approval of the US Food and Drug Administration (FDA) and the European Medicines Agency (EMEA) [23].

### 2.2. Echinocandins: Inhibitors of the Glucan Synthesis

Echinocandins (caspofungin, micafungin, and anidulafungin) are lipopeptidic antifungal agents that inhibit the synthesis of fungal wall by noncompetitive blockage of the (1,3)-*β*-D-glucan synthase (Figure 1). This enzyme inhibition leads to the formation of fungal cell walls with impaired structural integrity, which finally results in cell vulnerability to osmotic lysis [24]. All three agents (caspofungin, micafungin, and anidulafungin) exhibit concentration-dependent fungicidal activity against most species of *Candida* [25, 26] and have been approved by the regulatory agency FDA for the treatment of esophageal and invasive candidiasis, including candidemia [27–29].

### 2.3. Polyenes: Binding Ergosterol

Polyenes such as nystatin and amphotericin B (both isolated from *Streptomyces *spp.) bind ergosterol and disrupt the major lipidic component of the fungal cell membrane resulting in the production of aqueous pores (Figure 1). Consequently, the cellular permeability is altered and leads to the leakage of cytosolic components and, therefore, fungal death [30].

### 2.4. Nucleoside Analogues: Inhibitors of DNA/RNA Synthesis

Flucytosine is a pyrimidine analogue. It is transported into fungal cells by cytosine permeases. Then, it is deaminated to 5-fluorouracil and phosphorylated to 5-fluorodeoxyuridine monophosphate. This fluorinated nucleotide inhibits thymidylate synthase and thus interferes with DNA synthesis (Figure 1, [31]). The 5-fluorodeoxyuridine monophosphate can be further phosphorylated and incorporated to RNA, thus affecting RNA and protein synthesis (Figure 1, [32]).

### 2.5. Other Antifungal Agents

Allylamines and thiocarbamates also disrupt the cell membrane by inhibiting the squalene-epoxidase [33], enzyme involved in the biosynthesis of ergosterol (Figure 1). 

Griseofulvin (a tricyclic spirodiketone, first isolated from *Penicillium griseofulvum*) acts by disrupting spindle and cytoplasmic microtubule production, thereby inhibiting fungal mitosis (Figure 1, [34]).

### 2.6. Treatment of Systemic Infections

The antifungal therapy is driven by whether the agents are being used to treat mucosal or systemic infections. Superficial infections can be treated successfully with topical antifungal drugs. Systemic infections can be treated with oral or intravenous (IV) preparations.  Table 1 shows the pharmacokinetic parameters of the main antifungal agents used for the treatment of systemic candidiasis. Pharmacokinetic parameters are not always directly comparable because data derive from multiple sources and trials [20]. However, the routes of administration and excretion are often important considerations in selecting an appropriate antifungal agent. Some drugs are available only as IV preparations (e.g., caspofungin, micafungin, anidulafungin, and amphotericin B), only as oral preparations (e.g., posaconazole and flucytosine) or can be administered by both IV and oral routes (e.g., fluconazole, itraconazole, and voriconazole) depending on the drug solubility [35]. Since fluconazole and caspofungin are primarily excreted into the urine as active forms (Table 1), they are agents of choice for the treatment of urinary tract fungal infections. Unfortunately, some of these antifungal drugs have been extensively used and led to an increased selective pressure and the development of antifungal resistance [36]. 

## 3. Mechanisms of Resistance against Antifungal Agents

Antifungal resistance is based on different mechanisms, namely, (i) reduced drug intracellular accumulation, (ii) decreased target affinity/processivity for the drug, and (iii) counteraction of the drug effect. Particularly, the mechanism of resistance will be different depending on the mode of action of antifungal compounds. Cellular and molecular mechanisms supporting resistance against antifungal classes mentioned above have been discussed in detail in previous reviews [43–46]. Below, we briefly summarize the main observations (Table 2).

### 3.1. Azole Resistance

Over the past 10 years, fluconazole and itraconazole have been used extensively for chemoprophylaxis and treatment of systemic fungal infections because of their favorable oral bioavailability and safety profiles [84–86]. Afterwards, fluconazole resistance has been described in a high percentage of patients [87]. In fact, azole-resistant *C. albicans *is frequent in HIV-infected patients with oropharyngeal candidiasis [88]. However, resistance is less important in patients with other diseases, such as vaginal candidiasis and candidemia [89]. An intrinsically reduced susceptibility to fluconazole has been also reported for non-*albicans* species of *Candida* like *C. glabrata*, *C. krusei*, and *C. lusitaniae* [90, 91]. It appears that variations in the structure of azoles are responsible for the cross-resistance patterns among *Candida* species [92–94]. Several major mechanisms leading to azole resistance have been elucidated (Table 2, [95]) and detailed below.

(*i*) *Reduced Drug Intracellular Accumulation*. A responsible mechanism for decreasing the intracellular concentration of azole relies on an upregulation of two principal families of efflux pumps (reviewed in [96]). These transporters differ in the source of energy used to pump out the drug and in the specificity of the azole molecule. The Cdr pumps belong to the superfamily of ATP-binding cassette (ABC) transporters and are able to extrude all azole antifungals. These pumps are encoded by *Candida* drug resistance 1 and 2 (*CDR1* and *CDR2*) genes in *C. albicans* [96]. The other pump is a secondary transporter which utilizes proton gradient as a source of energy and is specific for fluconazole. This pump belongs to the major facilitator superfamily (MFS) transporters and is encoded by the *MDR1* gene in *C. albicans* [96]. Upregulation of *CDR1/CDR2 *and *MDR1 *arises from mutations in *TAC1* and *MRR1* transcription factors, respectively [97, 98]. Gain-of-function mutations generate hyperactive alleles in *C. albicans* and subsequent loss of heterozigocyty (LOH) at the *TAC1* and *MRR1* loci [99]. Other transporter genes have been reported to be upregulated in azole-resistant *C. glabrata* (Cg*CDR1*, Cg*CDR2* (formerly named *PDH1*) and Cg*SNQ2* (another ABC transporter)) [100–102], *C. dubliniensis* (Cd*CDR1* and Cd*CDR2*) [103], *C. krusei* (ABC1 and 2) [104, 105], and *C. tropicalis* (CDR1-homologue) isolates [44]. In *C. glabrata*, Cg*CDR1*, Cg*CDR2,* and Cg*SNQ2* genes are regulated by the Cg*PDR1* transcription factor [106–108]. 

(*ii*)  *Decreased Target Affinity for the Drug*. The target of azole antifungals is the lanosterol 14-*α*-demethylase encoded by the *ERG11* gene. Several point mutations have been characterized and associated to azole minimum inhibitory concentration (MIC) increases (reviewed in [95]). 

(*iii*) *Counteraction of the Drug Effect*. Two mechanisms contribute to counterbalancing the drug effects. The first system involves an upregulation of the *ERG11* gene leading to an intracellular increase of the target protein. ERG11 overexpression occurs by transcription factor regulation and gene duplication (reviewed in [95]). The second mechanism, although very uncommon, has been identified in several clinical isolates of *C. albicans* [109]. Alteration of the late steps of the biosynthesis of ergosterol through *ERG3 *inactivation leads to the total inactivation of the C5 sterol desaturase [110]. Thus, toxic 14*α*-methylated sterols are no longer accumulated, and yeast strains produce cell membranes devoid of ergosterol but containing other sterols [110].

### 3.2. Echinocandin Resistance

Echinocandin drugs are recommended as the first line for invasive candidiasis. However, reports of echinocandin resistance in patients with infections due to *C. albicans*, *C. glabrata*, *C. tropicalis,* and *C. krusei* are rising [111–116]. In fact, resistance in *C. glabrata* increased from 4.9% to 12.3% between 2001 and 2010 [115]. Even more, emergence of coresistance to both echinocandins and azoles in clinical isolates of *C. glabrata* has been reported [115]. In addition, intrinsic echinocandin resistance of *C. parapsilosis, C. orthopsilosis*, *C. metapsilosis,* and *C. guilliermondii* has been described [117, 118]. 

Secondary resistance to echinocandins is associated with the following mechanism. 

(*ii*)  *Decreased Target Processivity for the Drug*. Resistance is attributed to point mutations in the *FKS1* and/or *FKS2* genes (Table 2, [119–121]) which encode the (1,3)-*β*-D-glucan synthase complex [121]. Mutations in *FKS1* did not alter substrate binding but lowered *V*_max⁡_ values [122].

### 3.3. Polyene Resistance

Despite more than 30 years of clinical use, minimal resistance to amphotericin B has been developed. However, the main problem associated with the prophylactic use of conventional amphotericin B has always been due to its well-known side effects and toxicity [123, 124]. Resistance tends to be species dependent. *C. glabrata *and *C. krusei *are usually considered to be susceptible to amphotericin B, although they show higher MICs to polyenes than *C. albicans. *In this regard, higher than usual doses of amphotericin B have been recommended by the Infectious Diseases Society of America for treating candidemia caused by *C. glabrata *and *C. krusei* [125]. In fact, a significant proportion of isolates of *C. glabrata *and *C. krusei* species resistant to amphotericin B has been reported [126]. Additionally, some *Candida* spp. including *C. lusitaniae* and *C. guilliermondii*, besides *C. glabrata*, are capable of expressing resistance to amphotericin B [127]. It is noteworthy that even the antifungal lipopeptide caspofungin led to drug resistance in transplanted patients [112]. When resistance to polyenes occurs, it may result from the following mechanism.

(*iii*) *Counteraction of the Drug Effect*. Acquired resistance is probably due to a decrease or lack of ergosterol content in cell membranes. In fact, membranes of polyene-resistant *Candida* isolates have relatively low ergosterol content, compared to those of polyene-susceptible isolates. These deficiencies are probably consequences of loss of function mutations in the *ERG3* or *ERG6* genes which encode some of the enzymes involved in ergosterol biosynthesis (Table 2, [128–130]). 

### 3.4. Flucytosine Resistance

Primary resistance to flucytosine remains low (<2%). Secondary resistance relies on inactivation of different enzymes of the pyrimidine pathway (Table 2) as described below. 

(*i*) *Reduced Drug Intracellular Accumulation*. Uptake of the drug is affected by point mutations in the *FCY2* gene which encodes the cytosine permease [46, 128].

(*iii*) *Counteraction of the Drug Effect*. Acquired resistance to flucytosine also results from point mutations in the *FCY1* gene which encodes for the cytosine deaminase or *FUR1* gene which encodes for the uracil phosphoribosyl transferase. These enzymes catalyze the conversion of 5-fluorocytosine to 5-fluorouracil and 5-fluorouracil to 5-fluorouridine monophosphate, respectively. The most frequently acquired resistance to flucytosine is based on point mutations in the *FUR1* gene. Several point mutations have been described in *C. albicans*, *C. glabrata,* and *C. lusitaniae* [46, 128, 131, 132].

The rapid development of antifungal resistance, the toxicity and the variability in available formulations of some agents, and the increase in the frequency of non-*albicans Candida* spp. infections support the need for more effective and less toxic treatment strategies.

## 4. **Need of New Antifungal Agents**

Potential pharmacological strategies include the use of (i) new formulations of antifungals, such as liposomal amphotericin B, amphotericin B lipid complex, amphotericin B colloidal dispersion, amphotericin B into a lipid nanosphere formulation, itraconazole, and *β*-cyclodextrin itraconazole or (ii) combination therapies of one or more antifungal compounds, for example, amphotericin B + flucytosine, fluconazole + flucytosine, amphotericin B + fluconazole, caspofungin + liposomal amphotericin B, and caspofungin + fluconazole*.*

Potential alternative therapies include the use of new active principles obtained from different general sources, as natural products, synthetic agents or polymeric materials that have been shown to be active *in vitro* (Table 3). Among the natural products, plants contain diverse components that are important sources of biologically active molecules [50, 133, 134]. In fact, the activity of plant crude extracts against different microorganisms has been reported, that is, strong antifungal activity of some major components of essential oils [135, 136]. In this regard, the antibiofilm activity of terpenes and the exceptional efficiency of carvacrol, geraniol, and thymol, in the treatment of candidiasis associated with medical devices, have been demonstrated [137]. In another work, terpenoids exhibited excellent activity against *C. albicans* yeast and hyphal form growth at concentrations that were nontoxic to HeLa cells [138]. Thus, terpenoids may be useful in the near future not only as an antifungal chemotherapeutic agent but also to synergize effects of conventional drugs like fluconazole [138]. Other compounds with antimycological activity obtained from plants are saponins, alkaloids, peptides, and proteins [47, 48]. Marine organisms, endophytic fungi and microorganisms of terrestrial environment are also specific sources of antifungal compounds, although to a lesser extent [50, 139]. Among them, good antimicrobial activities of anthracycline-related compounds, peptides, pyrones, lipopeptides, and terpenoids isolated from these specific sources have been reported [49–51]. 

A second general source of antifungal agents comprises nonpolymeric synthetic agents, which can be classified into four groups (Table 3). The first group includes chemicals based on N,N-dimethylbiguanide complexes [52]. These compounds displayed low cytotoxicity and could be considered as potential broad-spectrum agents [53]. The second group involves derived compounds of traditional antifungal structures [54, 55] where some of them present better antimicrobial action than the original structures [55, 140]. The third group is formed by synthetic derived peptides, that is, the “human lactoferrin derived peptide” which was well tolerated in preclinical tests and clinical trials [56]. Finally, the last group includes compounds which are derived from semisynthetic natural products, such as compounds derived from echinocandins: micafungin sodium, anidulafungin, caspofungin acetate, and pneumocandin. These agents showed improved properties over the parental compounds [50, 141]. Unfortunately, echinocandins derivatives are poorly absorbed when administered orally and, therefore, are used only for IV administration. A natural antifungal with comparable activity to that of caspofungin acetate against *Candida* pathogenic fungal strains was isolated [51]. The compound, named enfumafungin, is a new triterpene glycoside that inhibits the (1,3)-*β*-D-glucan synthase. Several synthetic products derived from enfumafungin are currently under development in order to optimize *in vivo* antifungal activity and oral efficacy [57]. 

The third general source of antifungal compounds, namely, polymeric materials could be classified into seven groups (Table 3). (1) Polymers with quaternary nitrogen atoms [60] that can exist in different structures, that is, aromatic or heterocyclic structures [59], cationic conjugated polyelectrolytes [58], quaternary nitrogen atoms within the main chain [60], block copolymers [61], and synthetic and dendrimeric peptides [62, 63]. All of them were shown to be effective against a variety of microorganisms based on the exposure of its quaternary ammonium group. (2) Mimic antimicrobial peptides; among them are arylamide and phenylene ethynylene backbone polymers [64]*; *polynorbornene derivatives, which depending on their structure may exhibit substantial antimicrobial and low hemolytic activity [65], and polymethacrylate and polymethacrylamide with hydrophobic and cationic side chains [66, 67]. (3) Polymers with antimicrobial activity derived from their superficial activity (surfactants) based on fluorine-containing compounds [68]. (4) Polymers containing different contents of halogens, where the halogen group is the commander of the inhibition process, such as phenyl methacrylate polymers with different contents of chlorine [69, 70]. The halogen may form a covalent bond to nitrogen yielding polymeric N-halamines with a broad-spectrum antimicrobial activity without causing environmental concerns [71]. (5) Chelates; the antimicrobial activity of different chelates, such as N-vinylimidazole copolymerized with phenacyl methacrylate or poly (1,3-thiazol-2-yl-carbamoyl) methyl methacrylate with Cd(II), Cu(II), or Ni (II), has been analyzed in 2011 by Soykan et al. [73]. The Ni(II) complexes showed higher activity than those of Cu(II) and Co(II) ions. However, all of them exhibited lower activity than fluconazole. Another complex containing Cu(II) was found to have good antifungal activity due to electrostatic binding to fungal DNA [72]. (6) Imidazole derivatives, polymers and copolymers, with antimicrobial effectiveness depending on the polymeric structures [71, 142]. (7) Polymers loaded with antimicrobial organic or inorganic compounds. Antimicrobial organic agents are based on organic drugs; that is, chlorhexidine has been incorporated into polymeric microparticles and into polymeric hydrogels to modulate the release of the drug [74, 75]. Another research group loaded triclosan into polymeric nanoparticles [76]. Antimicrobial inorganic agents frequently incorporate metals into polymers, such as silver. This metal exhibits much higher toxicity to microorganisms than to mammalian cells. Polymeric nanotubes [77] and nanofibers [78] with silver nanoparticles have been prepared by chemical oxidation polymerization of rhodanine. Other silver nanocomposites have been reported in the literature based on different silver-loaded nanoparticles such as silver-zirconium phosphate nanoparticles [79] or silver zeolites [142]. Another example of inorganic compound loaded into polymers is copper. Copper particles are also known for their antimicrobial activity, although they are relatively less studied than silver [143]. 

**Figure 2:**
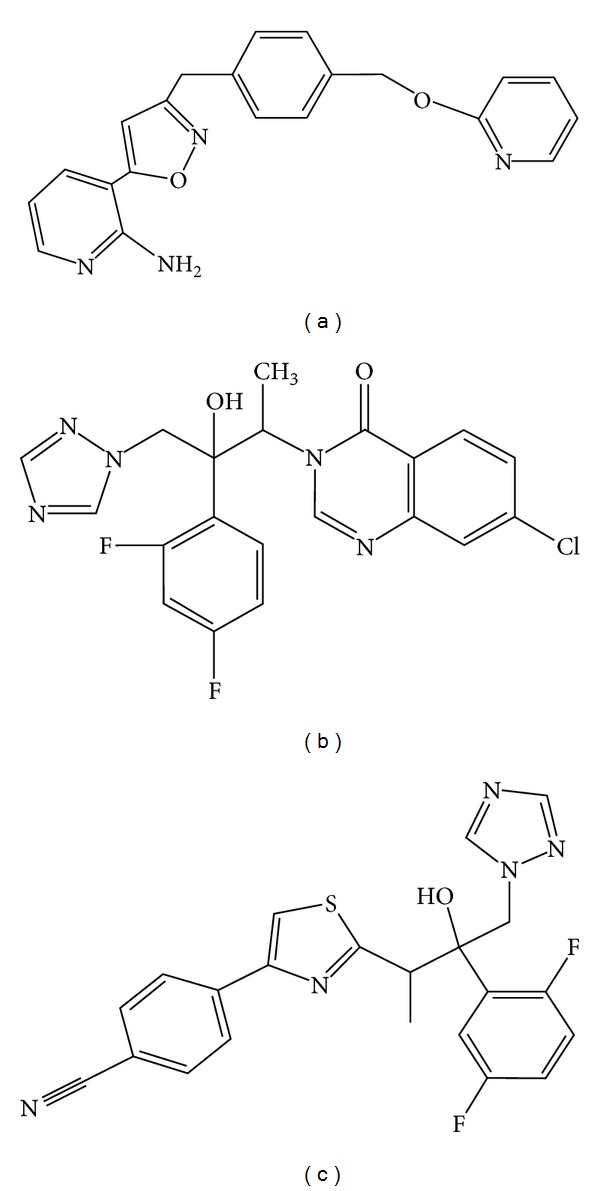
Chemical structures of three agents with actual promise: E1210 (a), albaconazole (b), and isavuconazole (c).

The mentioned agents have been tried *in vitro* against *Candida*; however, many of them are not used in clinical treatments; in this regard, there are three agents with actual promise: E1210, albaconazole, and isavuconazole (Figure 2).

E1210 is a broad-spectrum antifungal agent with a novel mechanism of action based on the inhibition of fungal glycosylphosphatidylinositol biosynthesis [144, 145]. The efficacy of oral E1210 was evaluated in murine models of oropharyngeal and disseminated candidiasis [80]. 

Results indicate that E1210 significantly reduced the number of viable *Candida* in the oral cavity in comparison to that of the control treatment and prolonged survival of mice infected with *Candida* spp. Therapeutic responses were dose dependent [80].  Table 4 shows the major pharmacokinetic parameters after administration of E1210 in mice. E1210 was also highly effective in the treatment of disseminated candidiasis caused by azole-resistant *C. albicans* or *C. tropicalis* [80]. Currently, E1210 is in Phase II.

Albaconazole is a new oral triazole with broad-spectrum antifungal activity, unique pharmacokinetics, and excellent tolerability [146]. It has been demonstrated that this compound was highly effective *in vitro *against pathogenic yeasts and also in animal models of systemic candidiasis [146]. Oral bioavailability was calculated to be 80% in rats and 100% in dogs [81]. Assays in healthy human volunteers showed that albaconazole was rapidly absorbed and presented good pharmacokinetic parameters (Table 4). In fact, the therapeutic efficacy of a single dose of albaconazole at ≥40 mg was more effective than 150 mg of fluconazole for the treatment of acute vulvovaginal candidiasis [81]. Currently, albaconazole is in Phase II. In addition, low toxicity was observed when albaconazole was administered to animals and human volunteers [82].

Finally, isavuconazole (the active metabolite of the water-soluble prodrug isavuconazonium) is a novel second-generation water-soluble triazole with broad-spectrum antifungal activity, also against azole-resistant strains. Studies carried out with neutropenic mice of disseminated *C. tropicalis* or *C. krusei* infections showed that the treatment significantly reduced kidney burden in mice infected with *C. tropicalis* and both kidney and brain burden in mice infected with *C. krusei* [147]. This azole is currently under Phase III trials in patients with systemic candidiasis. Both oral and intravenous formulations showed favorable pharmacokinetic (Table 4) and pharmacodynamic profiles [82]. This drug has the potential to become an important agent for the treatment of invasive fungal infections, principally because of its relatively broad and potent *in vitro* antifungal activity, its favorable pharmacokinetic profile, and the absence of severe adverse effects [82, 148, 149]. 

## 5. Conclusions

Although the antifungal drugs used in clinical treatments appear to be diverse and numerous, only few classes of antifungal agents are currently available in oral and intravenous forms. Additionally antifungal resistance based on different mechanisms continues to grow and evolve and exacerbate the need of new treatments against *Candida* infections. In this regard, new formulations of antifungals, combination therapies and development of new bioactive compounds might be useful for a better therapeutic outcome. Particularly, there are three compounds in Phase II or III studies with actual promise for the treatment of invasive candidiasis.

## Figures and Tables

**Table 1:** Administration routes and pharmacokinetic parameters of representative antifungal agents belonging to the major families of compounds.

Drug family	Drug	Adm. route^a^	Pharmacokinetic parameters						References
			Oral bioavailability (%)	*C*_max⁡_^b^ *μ*g/mL	AUC^c^ mg·h/L	Protein binding (%)	Half time (h)	Elimination	
Azoles	Fluconazole	Oral	>90	0.7	400.0	10–12	27–31	Urine	[35, 37]
	Itraconazole	Oral	>55	1.1	29.2	99.8	21–64	Hepatic	[35, 37]
	Voriconazole	Oral	>90	4.6	20.3	60.0	6	Renal	[35, 37, 38]
	Posaconazole	Oral	>98	7.8	17.0	99.0	15–35	Feces	[35, 39]

Echinocandins	Caspofungin	IV	<5	9.5–12.1	93.5–100.5	96.0	10.6	Urine	[20, 35, 40, 41]
	Micafungin	IV	<5	7.1–10.9	59.9–111.3	99.8	11–17	Feces	[20, 35, 40, 41]
	Anidulafungin	IV	<5	3.4–7.5	44.4–104.5	84.0	18.1–25.6	Feces	[20, 35, 40, 41]

Polyenes	Amphotericin B	IV	<5	1.5–2.1	13–17	>95	6.8–50	Feces	[35, 42]

Nucleoside analogues	Flucytosine	Oral	76–89	80	62	4	3–6	Renal	[31, 35]

^a^Adm. route indicates administration route; fluconazole, itraconazole, and voriconazole can be administered by both intravenous and oral routes; IV: intravenous; ^b^ *C*_max⁡_: maximal concentration; ^c^AUC: area under the curve.

**Table 2:** Resistance mechanisms of major systemic antifungal drugs. Antifungal resistance is based on different mechanisms, namely, (i) reduced drug intracellular accumulation, (ii) decreased target affinity/processivity for the drug, and (iii) counteraction of the drug effect.

Antifungal class	Genetic basis for resistance	Functional basis for resistance
Azoles	Upregulation of *CDR1*/*CDR2* and *MDR1 *by point mutations in *TAC1* and *MRR1* transcription factors	(i) Upregulation of drug transporters
	Point mutations in *ERG11 *	(ii) Decreased lanosterol 14-*α*-demethylase binding affinity for the drug
	Upregulation of *ERG11* by gene duplication and transcription factor regulation	(iii) Increased concentration of lanosterol 14-*α*-demethylase
	Point mutations in *ERG3 *	(iii) Inactivation of C5 sterol desaturase leading to alterations in the ergosterol synthetic pathway

Echinocandins	Point mutations in *FKS1* and *FKS2 *	(ii) Decreased glucan synthase processivity for the drug

Polyenes	Point mutations in *ERG3* and *ERG6 *	(iii) Decreased ergosterol content in cells

Nucleoside analogues	Point mutations in *FCY2 *	(i) Inactivation of cytosine permease affecting drug uptake
	Point mutations in *FCY1 *	(iii) Inactivation of cytosine deaminase leading to alterations in the metabolism of 5-fluorocytosine
	Point mutations in *FUR1 *	(iii) Inactivation of uracil phosphoribosyl transferase leading to alterations in the metabolism of 5-fluorocytosine

**Table 3:** Some natural products, synthetic agents, and polymeric materials with reported antifungal activities.

General source	Specific source	Biological active molecules	Examples	References
Natural products	Plants	Essential oils; terpenoids; saponins; phenolic compounds; alkaloids; peptides; proteins	Steroidal saponins, sesquiterpenoids	[47, 48]
	Marine organisms	Anthracycline-related compounds; lipopeptides; pentacyclic compounds	Xestodecalactone B, seragikinone A	[49]
	Endophytic fungi	Secondary metabolites; peptides; pyrones	cryptocandin, pestalopyrone	[50]
	Microorganisms of terrestrial environment	Lipopeptides; terpenoids	Echinocandins, enfumafungin	[50, 51]

Synthetic agents	Organically synthesized or derived compounds (not polymeric materials)	Compounds based on N,N-dimethylbiguanide complexes	Me (N,N-dimethylbiguanide)_2_(CH_3_COO)_2_·*n*H_2_O where Me: Mn, Ni, Cu, and Zn	[52, 53]
		Derived compounds from traditional antifungal structures	Imidazole derivatives, amine-derived bis-azoles	[54, 55]
		Synthetic derived peptides	Lactoferrin-derived peptides	[56]
		Derived compounds from natural products	Micafungin sodium, anidulafungin, caspofungin acetate, pneumocandin, and enfumafungin derivatives	[57, 58]

Polymeric materials	Polymeric materials	Polymers with quaternary nitrogen atoms	Polymers containing aromatic or heterocyclic structures	[59]
			Cationic conjugated polyelectrolytes	[58]
			Polymers with quaternary nitrogen atoms within the main chain.	[60]
			Block copolymers containing quaternary ammonium salt	[61]
			Synthetic peptides, synthetic dendrimeric peptides	[62, 63]
		Antifungal peptides mimics	Arylamide and phenylene ethynylene backbone polymers	[64]
			Polynorbornene derivatives	[65]
			Polymethacrylate and polymethacrylamide platforms containing hydrophobic and cationic side chains	[66, 67]
		Polymers with superficial activity	Fluorine-containing polymers	[68]
		Polymers containing different contents of halogens	Chlorine-containing phenyl methacrylate polymers	[69, 70]
			Polymeric N-halamines	[71]
		Chelates	Polymer-copper(II)-bipyridyl complex	[72]
			N-vinylimidazole copolymerized with phenacyl methacrylate	[73]
		Imidazole derivative polymers	2-[(5-methylisoxazol-3-yl)amino]-2-oxo-ethyl methacrylate and ethyl methacrylate	[71]
		Polymers loaded with antifungal compounds	Organic compounds	[74–76]
			Inorganic compounds	[77–79]

**Table 4:** Pharmacokinetic parameters of some lead drugs.

Drug	Available forms	Experimental organisms	Pharmacokinetic parameters						References
			Oral bioavailability (%)	*C*_max⁡_^b^ (*μ*g/mL)	*t*_max⁡_^c^ (h)	Protein binding (%)	Half time (h)	Elimination	
E1210	Oral/IV^a^	Mice	57.5	0.11	0.5	High	2.2	nr^d^	[80]
Albaconazole	Oral	Healthy human volunteers	nr^d^	5–80 (proportional to dose)	2–4	98	30–56	Feces	[81, 82]
Isavuconazonium	Oral	Healthy human volunteers	Very high	1.03 (100 mg dose)	0.75–1	98	56–77	Feces	[81–83]
Isavuconazole	IV^a^	Healthy human volunteers	nr^d^	1.45 (100 mg dose)	1.3–5	98	76–104	Feces	[81–83]

^a^IV: intravenous; ^b^ *C*_max⁡_: maximal concentration; ^c^ *t*_max⁡_: time to reach maximal plasma concentrations after oral administration; ^d^nr: not reported.

## References

[1] Jackson BE, Wilhelmus KR, Mitchell BM (2007). Genetically regulated filamentation contributes to *Candida albicans* virulence during corneal infection. *Microbial Pathogenesis*.

[2] Wu TG, Mitchell BM, Carothers TS (2003). Molecular analysis of the pediatric ocular surface for fungi. *Current Eye Research*.

[3] Achkar JM, Fries BC (2010). Candida infections of the genitourinary tract. *Clinical Microbiology Reviews*.

[4] Rosenbach A, Dignard D, Pierce JV, Whiteway M, Kumamoto CA (2010). Adaptations of *Candida albicans* for growth in the mammalian intestinal tract. *Eukaryotic Cell*.

[5] Naglik JR, Moyes DL, Wächtler B, Hube B (2011). *Candida albicans* interactions with epithelial cells and mucosal immunity. *Microbes and Infection*.

[6] López-Martínez R (2010). Candidosis, a new challenge. *Clinics in Dermatology*.

[7] Kontoyiannis DP, Mantadakis E, Samonis G (2003). Systemic mycoses in the immunocompromised host: an update in antifungal therapy. *Journal of Hospital Infection*.

[8] Zaoutis TE, Argon J, Chu J, Berlin JA, Walsh TJ, Feudtner C (2005). The epidemiology and attributable outcomes of candidemia in adults and children hospitalized in the United States: a propensity analysis. *Clinical Infectious Diseases*.

[9] Sydnor ERM, Perl TM (2011). Hospital epidemiology and infection control in acute-care settings. *Clinical Microbiology Reviews*.

[10] Pfaller MA, Diekema DJ (2004). Rare and emerging opportunistic fungal pathogens: concern for resistance beyond *Candida albicans* and *Aspergillus fumigatus*. *Journal of Clinical Microbiology*.

[11] Mikulska M, del Bono V, Ratto S, Viscoli C (2012). Occurrence, presentation and treatment of candidemia. *Expert Review of Clinical Immunology*.

[12] MacCallum DM (2012). Hosting infection: experimental models to assay Candida virulence. *International Journal of Microbiology*.

[13] Pfaller MA, Diekema DJ (2007). Epidemiology of invasive candidiasis: a persistent public health problem. *Clinical Microbiology Reviews*.

[14] Miceli MH, Díaz JA, Lee SA (2011). Emerging opportunistic yeast infections. *The Lancet Infectious Diseases*.

[15] Wisplinghoff H, Bischoff T, Tallent SM, Seifert H, Wenzel RP, Edmond MB (2004). Nosocomial bloodstream infections in US hospitals: analysis of 24,179 cases from a prospective nationwide surveillance study. *Clinical Infectious Diseases*.

[16] Cheng M-F, Yang Y-L, Yao T-J (2005). Risk factors for fatal candidemia caused by *Candida albicans* and non-albicans *Candida species*. *BMC Infectious Diseases*.

[17] Morrell M, Fraser VJ, Kollef MH (2005). Delaying the empiric treatment of Candida bloodstream infection until positive blood culture results are obtained: a potential risk factor for hospital mortality. *Antimicrobial Agents and Chemotherapy*.

[18] Mathew BP, Nath M (2009). Recent approaches to antifungal therapy for invasive mycoses. *ChemMedChem*.

[19] Kathiravan MK, Salake AB, Chothe AS (2012). The biology and chemistry of antifungal agents: a review. *Bioorganic & Medicinal Chemistry*.

[20] Denning DW, Hope WW (2010). Therapy for fungal diseases: opportunities and priorities. *Trends in Microbiology*.

[21] Hof H (2006). A new, broad-spectrum azole antifungal: posaconazole—mechanisms of action and resistance, spectrum of activity. *Mycoses*.

[22] Hay R, Katsambas A, Lotti T (2003). Antifungal drugs. *European Handbook of Dermatological Treatments*.

[23] Aparicio JF, Mendes MV, Antón N, Recio E, Martín JF (2004). Polyene macrolide antiobiotic biosynthesis. *Current Medicinal Chemistry*.

[24] Grover N (2010). Echinocandins: a ray of hope in antifungal drug therapy. *Indian Journal of Pharmacology*.

[25] Cappelletty D, Eiselstein-McKitrick K (2007). The echinocandins. *Pharmacotherapy*.

[26] Vazquez JA (2005). Anidulafungin: a new echinocandin with a novel profile. *Clinical Therapeutics*.

[27] Ostrosky-Zeichner L, Kontoyiannis D, Raffalli J (2005). International, open-label, noncomparative, clinical trial of micafungin alone and in combination for treatment of newly diagnosed and refractory candidemia. *European Journal of Clinical Microbiology and Infectious Diseases*.

[28] de Wet N, Llanos-Cuentas A, Suleiman J (2004). A randomized, double-blind, parallel-group, dose-response study of micafungin compared with fluconazole for the treatment of esophageal candidiasis in HIV-positive patients. *Clinical Infectious Diseases*.

[29] Mora-Duarte J, Betts R, Rotstein C (2002). Comparison of caspofungin and amphotericin B for invasive candidiasis. *The New England Journal of Medicine*.

[30] Sanglard D, Odds FC (2002). Resistance of *Candida species* to antifungal agents: molecular mechanisms and clinical consequences. *The Lancet Infectious Diseases*.

[31] Vermes A, Guchelaar H-J, Dankert J (2000). Flucytosine: a review of its pharmacology, clinical indications, pharmacokinetics, toxicity and drug interactions. *Journal of Antimicrobial Chemotherapy*.

[32] Onishi J, Meinz M, Thompson J (2000). Discovery of novel antifungal (1,3)-*β*-D-glucan synthase inhibitors. *Antimicrobial Agents and Chemotherapy*.

[33] Sanglard D, Coste A, Ferrari S (2009). Antifungal drug resistance mechanisms in fungal pathogens from the perspective of transcriptional gene regulation. *FEMS Yeast Research*.

[34] François IEJA, Aerts AM, Cammue BPA, Thevissen K (2005). Currently used antimycotics: spectrum, mode of action and resistance occurrence. *Current Drug Targets*.

[35] Ashley ESD, Lewis R, Lewis JS, Martin C, Andes D (2006). Pharmacology of systemic antifungal agents. *Clinical Infectious Diseases*.

[36] Espinel-Ingroff A (2009). Novel antifungal agents, targets or therapeutic strategies for the treatment of invasive fungal diseases: a review of the literature (2005–2009). *Revista Iberoamericana de Micologia*.

[37] Lipp H-P (2010). Clinical pharmacodynamics and pharmacokinetics of the antifungal extended-spectrum triazole posaconazole: an overview. *British Journal of Clinical Pharmacology*.

[38] Theuretzbacher U, Ihle F, Derendorf H (2006). Pharmacokinetic/pharmacodynamic profile of voriconazole. *Clinical Pharmacokinetics*.

[39] Li Y, Theuretzbacher U, Clancy CJ, Nguyen MH, Derendorf H (2010). Pharmacokinetic/pharmacodynamic profile of posaconazole. *Clinical Pharmacokinetics*.

[40] Theuretzbacher U (2004). Pharmacokinetics/pharmacodynamics of echinocandins. *European Journal of Clinical Microbiology and Infectious Diseases*.

[41] Wagner C, Graninger W, Presterl E, Joukhadar C (2006). The echinocandins: comparison of their pharmacokinetics, pharmacodynamics and clinical applications. *Pharmacology*.

[42] Bekersky I, Fielding RM, Dressler DE, Lee JW, Buell DN, Walsh TJ (2002). Pharmacokinetics, excretion, and mass balance of liposomal amphotericin B (AmBisome) and amphotericin B deoxycholate in humans. *Antimicrobial Agents and Chemotherapy*.

[43] Kanafani ZA, Perfect JR (2008). Resistance to antifungal agents: mechanisms and clinical impact. *Clinical Infectious Diseases*.

[44] Vandeputte P, Ferrari S, Coste AT (2012). Antifungal resistance and new strategies to control fungal infections. *International Journal of Microbiology*.

[45] Perlin DS (2009). Antifungal drug resistance: do molecular methods provide a way forward?. *Current Opinion in Infectious Diseases*.

[46] Pemán J, Cantón E, Espinel-Ingroff A (2009). Antifungal drug resistance mechanisms. *Expert Review of Anti-Infective Therapy*.

[47] Abad MJ, Ansuategui M, Bermejo P (2007). Active antifungal substances from natural sources. *Arkivoc*.

[48] Li Y-Y, Hu Z-Y, Lu C-H, Shen Y-M (2010). Four new terpenoids from Xylaria sp. 101. *Helvetica Chimica Acta*.

[49] Bhadury P, Mohammad BT, Wright PC (2006). The current status of natural products from marine fungi and their potential as anti-infective agents. *Journal of Industrial Microbiology and Biotechnology*.

[50] Sortino M, Derita M, Svetaz L, Filho VC (2012). 6. The role of natural products in discovery of new anti-infective agents with emphasis on antifungal compounds. *Plant Bioactives and Drug Discovery: Principles, Practice, and Perspectives*.

[51] Peláez F, Cabello A, Platas G (2000). The discovery of enfumafungin, a novel antifungal compound produced by an endophytic Hormonema species biological activity and taxonomy of the producing organisms. *Systematic and Applied Microbiology*.

[52] Olar R, Badea M, Marinescu D (2010). Prospects for new antimicrobials based on N,N-dimethylbiguanide complexes as effective agents on both planktonic and adhered microbial strains. *European Journal of Medicinal Chemistry*.

[53] Olar R, Badea M, Marinescu D (2010). N, N-dimethylbiguanide complexes displaying low cytotoxicity as potential large spectrum antimicrobial agents. *European Journal of Medicinal Chemistry*.

[54] Anderson EB, Long TE (2010). Imidazole- and imidazolium-containing polymers for biology and material science applications. *Polymer*.

[55] Fang B, Zhou C-H, Rao X-C (2010). Synthesis and biological activities of novel amine-derived bis-azoles as potential antibacterial and antifungal agents. *European Journal of Medicinal Chemistry*.

[56] Brouwer CPJM, Rahman M, Welling MM (2011). Discovery and development of a synthetic peptide derived from lactoferrin for clinical use. *Peptides*.

[57] Heasley BH, Pacofsky GJ, Mamai A (2012). Synthesis and biological evaluation of antifungal derivatives of enfumafungin as orally bioavailable inhibitors of *β*-1, 3-glucan synthase. *Bioorganic & Medicinal Chemistry Letters*.

[58] Melo LD, Mamizuka EM, Carmona-Ribeiro AM (2010). Antimicrobial particles from cationic lipid and polyelectrolytes. *Langmuir*.

[59] Timofeeva LM, Kleshcheva NA, Moroz AF, Didenko LV (2009). Secondary and tertiary polydiallylammonium salts: novel polymers with high antimicrobial activity. *Biomacromolecules*.

[60] Cakmak I, Ulukanli Z, Tuzcu M, Karabuga S, Genctav K (2004). Synthesis and characterization of novel antimicrobial cationic polyelectrolytes. *European Polymer Journal*.

[61] Sauvet G, Fortuniak W, Kazmierski K, Chojnowski J (2003). Amphiphilic block and statistical siloxane copolymers with antimicrobial activity. *Journal of Polymer Science A*.

[62] Zhu J, Luther PW, Leng Q, Mixson AJ (2006). Synthetic histidine-rich peptides inhibit *Candida species* and other fungi in vitro: role of endocytosis and treatment implications. *Antimicrobial Agents and Chemotherapy*.

[63] Tam JP, Lu Y-A, Yang J-L (2002). Antimicrobial dendrimeric peptides. *European Journal of Biochemistry*.

[64] Tew GN, Clements D, Tang H, Arnt L, Scott RW (2006). Antimicrobial activity of an abiotic host defense peptide mimic. *Biochimica et Biophysica Acta*.

[65] Som A, Choi Y, Tew GN (2009). Monovalent salt effects on the membrane activity of antimicrobial polymers. *Macromolecular Symposia*.

[66] Palermo EF, Kuroda K (2009). Chemical structure of cationic groups in amphiphilic polymethacrylates modulates the antimicrobial and hemolytic activities. *Biomacromolecules*.

[67] Palermo EF, Sovadinova I, Kuroda K (2009). Structural determinants of antimicrobial activity and biocompatibility in membrane-disrupting methacrylamide random copolymers. *Biomacromolecules*.

[68] Caillier L, Taffin de Givenchy E, Levy R, Vandenberghe Y, Geribaldi S, Guittard F (2009). Polymerizable semi-fluorinated gemini surfactants designed for antimicrobial materials. *Journal of Colloid and Interface Science*.

[69] Patel MB, Patel SA, Ray A, Patel RM (2003). Synthesis, characterization, and antimicrobial activity of acrylic copolymers. *Journal of Applied Polymer Science*.

[70] Patel JN, Dolia MB, Patel KH, Patel RM (2006). Homopolymer of 4-chloro-3-methyl phenyl methacrylate and its copolymers with butyl methacrylate: synthesis, characterization, reactivity ratios and antimicrobial activity. *Journal of Polymer Research*.

[71] Muñoz-Bonilla A, Fernández-García M (2012). Polymeric materials with antimicrobial activity. *Progress in Polymer Science*.

[72] Senthil Kumar R, Sasikala K, Arunachalam S (2008). DNA interaction of some polymer-copper(II) complexes containing 2,2′-bipyridyl ligand and their antimicrobial activities. *Journal of Inorganic Biochemistry*.

[73] Soykan C, Coskun R, Kirbag S (2007). Poly(crotonic acid-co-2-acrylamido-2-methyl-1-propanesulfonic acid)-metal complexes with copper(II), cobalt(II), and nickel(II): synthesis, characterization and antimicrobial activity. *European Polymer Journal*.

[74] Yue IC, Poff J, Cortés ME (2004). A novel polymeric chlorhexidine delivery device for the treatment of periodontal disease. *Biomaterials*.

[75] Kiremitçi AS, Çiftçi A, Özalp M, Gümüşderelioğlu M (2007). Novel chlorhexidine releasing system developed from thermosensitive vinyl ether-based hydrogels. *Journal of Biomedical Materials Research B*.

[76] Zhang H, Wang D, Butler R (2008). Formation and enhanced biocidal activity of water-dispersable organic nanoparticles. *Nature Nanotechnology*.

[77] Kong H, Song J, Jang J (2009). One-step preparation of antimicrobial polyrhodanine nanotubes with silver nanoparticles. *Macromolecular Rapid Communications*.

[78] Kong H, Jang J (2008). Synthesis and antimicrobial properties of novel silver/polyrhodanine nanofibers. *Biomacromolecules*.

[79] Duan Y-Y, Jia J, Wang S-H, Yan W, Jin L, Wang Z-Y (2007). Preparation of antimicrobial poly(e-caprolactone) electrospun nanofibers containing silver-loaded zirconium phosphate nanoparticles. *Journal of Applied Polymer Science*.

[80] Hata K, Horii T, Miyazaki M (2011). Efficacy of oral E1210, a new broad-spectrum antifungal with a novel mechanism of action, in murine models of candidiasis, aspergillosis, and fusariosis. *Antimicrobial Agents and Chemotherapy*.

[81] Pasqualotto AC, Denning DW (2008). New and emerging treatments for fungal infections. *The Journal of Antimicrobial Chemotherapy*.

[82] Girmenia C (2009). New generation azole antifungals in clinical investigation. *Expert Opinion on Investigational Drugs*.

[83] Schmitt-Hoffmann A, Roos B, Heep M (2006). Single-ascending-dose pharmacokinetics and safety of the novel broad-spectrum antifungal triazole BAL4815 after intravenous infusions (50, 100, and 200 milligrams) and oral administrations (100, 200, and 400 milligrams) of its prodrug, BAL8557, in healthy volunteers. *Antimicrobial Agents and Chemotherapy*.

[84] Meis JFGM, Verweij PE (2001). Current management of fungal infections. *Drugs*.

[85] Hoffman HL, Ernst EJ, Klepser ME (2000). Novel triazole antifungal agents. *Expert Opinion on Investigational Drugs*.

[86] Livermore DM (2004). The need for new antibiotics. *Clinical Microbiology and Infection*.

[87] Redding SW, Kirkpatrick WR, Saville S (2003). Multiple patterns of resistance to fluconazole in *Candida glabrata* isolates from a patient with oropharyngeal candidiasis receiving head and neck radiation. *Journal of Clinical Microbiology*.

[88] Skiest DJ, Vazquez JA, Anstead GM (2007). Posaconazole for the treatment of azole-refractory oropharyngeal and esophageal candidiasis in subjects with HIV infection. *Clinical Infectious Diseases*.

[89] Ribeiro M, Paula CR, Perfect JR, Cox GM (2005). Phenotypic and genotypic evaluation of fluconazole resistance in vaginal Candida strains isolated from HIV-infected women from Brazil. *Medical Mycology*.

[90] Vazquez JA, Peng G, Sabel JO (2001). Evolution of antifungal susceptibility among *Candida species* isolates recovered from human immunodeficiency virus-infected women receiving fluconazole prophylaxis. *Clinical Infectious Diseases*.

[91] Safdar A, van Rhee F, Henslee-Downey JP, Singhal S, Mehta J (2001). *Candida glabrata* and *Candida krusei* fungemia after high-risk allogeneic marrow transplantation: no adverse effect of low-dose fluconazole prophylaxis on incidence and outcome. *Bone Marrow Transplantation*.

[92] Cuenca-Estrella M, Gomez-Lopez A, Mellado E, Buitrago MJ, Monzon A, Rodriguez-Tudela JL (2006). Head-to-head comparison of the activities of currently available antifungal agents against 3,378 Spanish clinical isolates of yeasts and filamentous fungi. *Antimicrobial Agents and Chemotherapy*.

[93] Pfaller MA, Messer SA, Boyken L (2007). Use of fluconazole as a surrogate marker to predict susceptibility and resistance to voriconazole among 13,338 clinical isolates of Candida spp. tested by clinical and laboratory standards institute-recommended broth microdilution methods. *Journal of Clinical Microbiology*.

[94] Pfaller MA, Messer SA, Boyken L (2004). Cross-resistance between fluconazole and ravuconazole and the use of fluconazole as a surrogate marker to predict susceptibility and resistance to ravuconazole among 12,796 clinical isolates of Candida spp. *Journal of Clinical Microbiology*.

[95] Noël T (2012). The cellular and molecular defense mechanisms of the Candida yeasts against azole antifungal drugs. *Journal de Mycologie Médicale*.

[96] Cannon RD, Lamping E, Holmes AR (2009). Efflux-mediated antifungal drug resistance. *Clinical Microbiology Reviews*.

[97] Coste AT, Karababa M, Ischer F, Bille J, Sanglard D (2004). *TAC1*, transcriptional activator of CDR genes, is a new transcription factor involved in the regulation of *Candida albicans* ABC transporters *CDR1* and *CDR2*. *Eukaryotic Cell*.

[98] Coste A, Turner V, Ischer F (2006). A mutation in TAC1p, a transcription factor regulating *CDR1* and *CDR2*, is coupled with loss of heterozygosity at chromosome 5 to mediate antifungal resistance in *Candida albicans*. *Genetics*.

[99] Coste AT, Crittin J, Bauser C, Rohde B, Sanglard D (2009). Functional analysis of cis-and trans-acting elements of the *Candida albicans CDR2*promoter with a novel promoter reporter system. *Eukaryotic Cell*.

[100] Torelli R, Posteraro B, Ferrari S (2008). The ATP-binding cassette transporter-encoding gene *CgSNQ2* is contributing to the CgPDR1-dependent azole resistance of *Candida glabrata*. *Molecular Microbiology*.

[101] Sanglard D, Ischer F, Calabrese D, Majcherczyk PA, Bille J (1999). The ATP binding cassette transporter gene *CgCDR1* from *Candida glabrata* is involved in the resistance of clinical isolates to azole antifungal agents. *Antimicrobial Agents and Chemotherapy*.

[102] Bennett JE, Izumikawa K, Marr KA (2004). Mechanism of Increased Fluconazole Resistance in *Candida glabrata* during Prophylaxis. *Antimicrobial Agents and Chemotherapy*.

[103] Moran GP, Sanglard D, Donnelly SM, Shanley DB, Sullivan DJ, Coleman DC (1998). Identification and expression of multidrug transporters responsible for fluconazole resistance in *Candida dubliniensis*. *Antimicrobial Agents and Chemotherapy*.

[104] Lamping E, Ranchod A, Nakamura K (2009). Abc1p is a multidrug efflux transporter that tips the balance in favor of innate azole resistance in *Candida krusei*. *Antimicrobial Agents and Chemotherapy*.

[105] Katiyar SK, Edlind TD (2001). Identification and expression of multidrug resistance-related ABC transporter genes in *Candida krusei*. *Medical Mycology*.

[106] Vermitsky J-P, Edlind TD (2004). Azole resistance in *Candida glabrata*: coordinate upregulation of multidrug transporters and evidence for a Pdr1-like transcription factor. *Antimicrobial Agents and Chemotherapy*.

[107] Vermitsky J-P, Earhart KD, Smith WL, Homayouni R, Edlind TD, Rogers PD (2006). Pdr1 regulates multidrug resistance in *Candida glabrata*: gene disruption and genome-wide expression studies. *Molecular Microbiology*.

[108] Tsai H-F, Krol AA, Sarti KE, Bennett JE (2006). *Candida glabrata PDR1* , a transcriptional regulator of a pleiotropic drug resistance network, mediates azole resistance in clinical isolates and petite mutants. *Antimicrobial Agents and Chemotherapy*.

[109] Martel CM, Parker JE, Bader O (2010). Identification and characterization of four azole-resistant erg3 mutants of *Candida albicans*. *Antimicrobial Agents and Chemotherapy*.

[110] Miyazaki Y, Geber A, Miyazaki H (1999). Cloning, sequencing, expression and allelic sequence diversity of *ERG3* (C-5 sterol desaturase gene) in *Candida albicans*. *Gene*.

[111] Hernandez S, López-Ribot JL, Najvar LK, McCarthy DI, Bocanegra R, Graybill JR (2004). Caspofungin resistance in *Candida albicans*: correlating clinical outcome with laboratory susceptibility testing of three isogenic isolates serially obtained from a patient with progressive candida esophagitis. *Antimicrobial Agents and Chemotherapy*.

[112] Krogh-Madsen M, Arendrup MC, Heslet L, Knudsen JD (2006). Amphotericin B and caspofungin resistance in *Candida glabrata* isolates recovered from a critically ill patient. *Clinical Infectious Diseases*.

[113] Hakki M, Staab JF, Marr KA (2006). Emergence of a *Candida krusei* isolate with reduced susceptibility to caspofungin during therapy. *Antimicrobial Agents and Chemotherapy*.

[114] Pasquale T, Tomada JR, Ghannoun M, Dipersio J, Bonilla H (2008). Emergence of *Candida tropicalis* resistant to caspofungin. *Journal of Antimicrobial Chemotherapy*.

[115] Alexander B, Johnson M, Pfeiffer C (2013). Increasing echinocandin resistance in *Candida glabrata*: clinical failure correlates with presence of *FKS* mutations and elevated minimum inhibitory concentrations. *Clinical Infectious Diseases*.

[116] Pfaller MA, Castanheira M, Lockhart SR, Ahlquist AM, Messer SA, Jones RN (2012). Frequency of decreased susceptibility and resistance to echinocandins among fluconazole-resistant bloodstream isolates of *Candida glabrata*. *Journal of Clinical Microbiology*.

[117] Garcia-Effron G, Katiyar SK, Park S, Edlind TD, Perlin DS (2008). A naturally occurring proline-to-alanine amino acid change in Fks1p in *Candida parapsilosis*, *Candida orthopsilosis*, and *Candida metapsilosis* accounts for reduced echinocandin susceptibility. *Antimicrobial Agents and Chemotherapy*.

[118] Cantón E, Pemán J, Sastre M, Romero M, Espinel-Ingroff A (2006). Killing kinetics of caspofungin, micafungin, and amphotericin B against *Candida guilliermondii*. *Antimicrobial Agents and Chemotherapy*.

[119] Kahn JN, Garcia-Effron G, Hsu M-J, Park S, Marr KA, Perlin DS (2007). Acquired echinocandin resistance in a *Candida krusei* isolate due to modification of glucan synthase. *Antimicrobial Agents and Chemotherapy*.

[120] Park S, Kelly R, Kahn JN (2005). Specific substitutions in the echinocandin target Fks1p account for reduced susceptibility of rare laboratory and clinical Candida sp. isolates. *Antimicrobial Agents and Chemotherapy*.

[121] Balashov SV, Park S, Perlin DS (2006). Assessing resistance to the echinocandin antifungal drug caspofungin in *Candida albicans* by profiling mutations in *FKS1*. *Antimicrobial Agents and Chemotherapy*.

[122] Garcia-Effron G, Park S, Perlin DS (2009). Correlating echinocandin MIC and kinetic inhibition of fks1 mutant glucan synthases for *Candida albicans*: implications for interpretive breakpoints. *Antimicrobial Agents and Chemotherapy*.

[123] Laniado-Laborín R, Cabrales-Vargas MN (2009). Amphotericin B: side effects and toxicity. *Revista Iberoamericana de Micologia*.

[124] Ellis D (2002). Amphotericin B: spectrum and resistance. *Journal of Antimicrobial Chemotherapy*.

[125] Rex JH, Walsh TJ, Sobel JD (2000). Practice guidelines for the treatment of candidiasis. *Clinical Infectious Diseases*.

[126] Kontoyiannis DP, Lewis RE (2002). Antifungal drug resistance of pathogenic fungi. *The Lancet*.

[127] Pappas PG, Rex JH, Sobel JD (2004). Guidelines for treatment of Candidiasis. *Clinical Infectious Diseases*.

[128] Espinel-Ingroff A (2008). Mechanisms of resistance to antifungal agents: yeasts and filamentous fungi. *Revista Iberoamericana de Micologia*.

[129] Vandeputte P, Tronchin G, Bergès T, Hennequin C, Chabasse D, Bouchara J-P (2007). Reduced susceptibility to polyenes associated with a missense mutation in the *ERG6* gene in a clinical isolate of *Candida glabrata* with pseudohyphal growth. *Antimicrobial Agents and Chemotherapy*.

[130] Kelly SL, Lamb DC, Kelly DE (1997). Resistance to fluconazole and cross-resistance to amphotericin B in *Candida albicans* from AIDS patients caused by defective sterol Δ5,6-desaturation. *FEBS Letters*.

[131] Chapeland-Leclerc F, Bouchoux J, Goumar A, Chastin C, Villard J, Noël T (2005). Inactivation of the *FCY2* gene encoding purine-cytosine permease promotes cross-resistance to flucytosine and fluconazole in *Candida lusitaniae*. *Antimicrobial Agents and Chemotherapy*.

[132] Vandeputte P, Pineau L, Larcher G (2011). Molecular mechanisms of resistance to 5-fluorocytosine in laboratory mutants of *Candida glabrata*. *Mycopathologia*.

[133] Duarte MCT, Figueira GM, Sartoratto A, Rehder VLG, Delarmelina C (2005). Anti-Candida activity of Brazilian medicinal plants. *Journal of Ethnopharmacology*.

[134] Butler MS (2004). The role of natural product chemistry in drug discovery. *Journal of Natural Products*.

[135] Mondello F, de Bernardis F, Girolamo A, Cassone A, Salvatore G (2006). In vivo activity of terpinen-4-ol, the main bioactive component of *Melaleuca alternifolia* Cheel (tea tree) oil against azole-susceptible and -resistant human pathogenic *Candida species*. *BMC Infectious Diseases*.

[136] Manohar V, Ingram C, Gray J (2001). Antifungal activities of origanum oil against *Candida albicans*. *Molecular and Cellular Biochemistry*.

[137] Dalleau S, Cateau E, Bergès T, Berjeaud J-M, Imbert C (2008). In vitro activity of terpenes against Candida biofilms. *International Journal of Antimicrobial Agents*.

[138] Zore GB, Thakre AD, Jadhav S, Karuppayil SM (2011). Terpenoids inhibit *Candida albicans* growth by affecting membrane integrity and arrest of cell cycle. *Phytomedicine*.

[139] Gunatilaka AAL (2006). Natural products from plant-associated microorganisms: distribution, structural diversity, bioactivity, and implications of their occurrence. *Journal of Natural Products*.

[140] Aher NG, Pore VS, Mishra NN (2009). Synthesis and antifungal activity of 1,2,3-triazole containing fluconazole analogues. *Bioorganic and Medicinal Chemistry Letters*.

[141] Kofla G, Ruhnke M (2011). Pharmacology and metabolism of anidulafungin, caspofungin and micafungin in the treatment of invasive candidosis—review of the literature. *European Journal of Medical Research*.

[142] Fernández A, Soriano E, Hernández-Muñoz P, Gavara R (2010). Migration of antimicrobial silver from composites of polylactide with silver zeolites. *Journal of Food Science*.

[143] Ozay O, Akcali A, Otkun MT, Silan C, Aktas N, Sahiner N (2010). P(4-VP) based nanoparticles and composites with dual action as antimicrobial materials. *Colloids and Surfaces B*.

[144] Watanabe N-A, Miyazaki M, Horii T, Sagane K, Tsukahara K, Hata K (2012). E1210, a new broad-spectrum antifungal, suppresses *Candida albicans* hyphal growth through inhibition of glycosylphosphatidylinositol biosynthesis. *Antimicrobial Agents and Chemotherapy*.

[145] Miyazaki M, Horii T, Hata K (2011). In vitro activity of E1210, a novel antifungal, against clinically important yeasts and molds. *Antimicrobial Agents and Chemotherapy*.

[146] Bartroli J, Merlos M (2011). Overview of albaconazole. *European Infectious Disease*.

[147] Majithiya J, Sharp A, Parmar A, Denning DW, Warn PA (2009). Efficacy of isavuconazole, voriconazole and fluconazole in temporarily neutropenic murine models of disseminated *Candida tropicalis* and *Candida krusei*. *Journal of Antimicrobial Chemotherapy*.

[148] Odds FC (2006). Drug evaluation: BAL-8557—a novel broad-spectrum triazole antifungal. *Current Opinion in Investigational Drugs*.

[149] Livermore J, Hope W (2012). Evaluation of the pharmacokinetics and clinical utility of isavuconazole for treatment of invasive fungal infections. *Expert Opinion on Drug Metabolism & Toxicology*.

